# Influence of the electrolyte’s pH on the properties of electrochemically deposited hydroxyapatite coating on additively manufactured Ti64 alloy

**DOI:** 10.1038/s41598-017-16985-z

**Published:** 2017-12-01

**Authors:** Alina Vladescu, Diana M. Vranceanu, Slawek Kulesza, Alexey N. Ivanov, Mirosław Bramowicz, Alexander S. Fedonnikov, Mariana Braic, Igor A. Norkin, Andrey Koptyug, Maria O. Kurtukova, Mihaela Dinu, Iulian Pana, Maria A. Surmeneva, Roman A. Surmenev, Cosmin M. Cotrut

**Affiliations:** 1 National Institute for Optoelectronics, Department for Advanced Surface Processing and Analysis by Vacuum Technologies, 409 Atomistilor St., Magurele, RO77125 Romania; 2 University Politehnica of Bucharest, 313 Spl. Independentei, Bucharest, RO60042 Romania; 3 Warmia and Mazury University in Olsztyn, Department of Mathematics and Computer Science, Słoneczna 54, Olsztyn, 10-719 Poland; 4 Scientific Research Institute of Traumatology, Orthopedics and Neurosurgery of Federal State Budgetary Educational Institution of Higher Education “V.I. Razumovsky Saratov State Medical University” of the Ministry of Healthcare of the Russian Federation, 148 Chernyshevskogo st., Saratov, 410012 Russia; 5 Additive Manufacturing Group, Sports Tech Research Centre, Mid Sweden University, Akademigatan 1, Östersund, 831 25 Sweden; 6 Department of Histology, Federal State Budgetary Educational Institution of Higher Education “V.I. Razumovsky Saratov State Medical University” of the Ministry of Healthcare of the Russian Federation, 112 Bolshaya Kazachia st., Saratov, 410012 Russia; 7 National Research Tomsk Polytechnic University, Lenin Avenue 43, Tomsk, 634050 Russia

## Abstract

Properties of the hydroxyapatite obtained by electrochemical assisted deposition (ED) are dependent on several factors including deposition temperature, electrolyte pH and concentrations, applied potential. All of these factors directly influence the morphology, stoichiometry, crystallinity, electrochemical behaviour, and particularly the coating thickness. Coating structure together with surface micro- and nano-scale topography significantly influence early stages of the implant bio-integration. The aim of this study is to analyse the effect of pH modification on the morphology, corrosion behaviour and *in vitro* bioactivity and *in vivo* biocompatibility of hydroxyapatite prepared by ED on the additively manufactured Ti64 samples. The coatings prepared in the electrolytes with pH = 6 have predominantly needle like morphology with the dimensions in the nanometric scale (~30 nm). Samples coated at pH = 6 demonstrated higher protection efficiency against the corrosive attack as compared to the ones coated at pH = 5 (~93% against 89%). The *in vitro* bioactivity results indicated that both coatings have a greater capacity of biomineralization, compared to the uncoated Ti64. Somehow, the coating deposited at pH = 6 exhibited good corrosion behaviour and high biomineralization ability. *In vivo* subcutaneous implantation of the coated samples into the white rats for up to 21 days with following histological studies showed no serious inflammatory process.

## Introduction

Tissue engineering is an interdisciplinary field aiming to produce medical case-specific biological substitutes that help circumvent the limitations of existing clinical treatment for the damaged tissue or organs. Efficient strategy to enhance the bioactivity of surfaces on advanced bioinspired materials involves the replication of the hierarchical organization and properties of biological tissue leading to novel functional constructions for medical applications. Different lattice and porous scaffolds are designed to mimic the architecture and functions of the natural extracellular matrix, providing a template for growth of the target tissue. The macrostructure feature of the bone can be modelled as a dense compact (cortical) outer shell with porous (cancellous) sections inside. Until recently scaffolds were mainly fabricated by solvent-casting particulate-leaching, gas foaming, fiber meshes/fiber bonding, phase separation, melt moulding, emulsion freeze drying, solution casting or freeze drying^1–3^. Listed methods have several limitations e.g. inadequate control over the pore dimensions, pore geometry and levels of interconnectivity, resulting in an impoverished cell distribution partly due to the inherent inaccuracies in the process of manual cell seeding^4,5^. Rapidly developing additive manufacturing (AM) technologies in metal and polymers (in popular literature these are often referred to as 3D printing) allowing for high manufacturing precision and ease of replication for complex-shaped structures provide a new way forward. These methods are capable of manufacturing components combining solid parts with both regular lattice type structures^6–8^ and irregular spongy ones^9,10^. *In vitro* cell experiments confirm that homogenous cell distribution beneficial for the regeneration of functional tissue can be obtained when using such structures^11–21^. The use of additive manufacturing to fabricate the 3D scaffolds mimicking natural bone structures also has a great potential for personalized medicine. Despite years of research, development of an ideal tissue engineering scaffold, which is crucial for the development of functional replacement for human tissues, has not been achieved yet. Though additive manufacturing today is capable of mimicking complex architecture of natural bone, it cannot yet achieve proper bio-functionalization with available materials (either metal or polymer) “as manufactured”. Two of the significant problems encountered are the composition and surface topography of implants as-manufactured by AM methods. With powder-bed fusion AM methods like EBM it is quite challenging to simultaneously control the tailored shape, surface composition, topography and surface roughness of the as manufactured components. There are process-related differences in the surface roughness and, in some cases, porosity and composition depending on the presence of certain structural elements (solid parts, thin ridges, lattices) and also the apparent angle of the surfaces to the build direction (overhanging surfaces are commonly rougher and more porous). Thus, a most promising next step is the post-processing of as additively manufactured implant structures including surface modifications and coating by advanced materials to improve their biocompatibility, stability and longevity in the human body.

Bioceramics-based coatings are today among the most promising candidates for improving the osseointegration of porous scaffolds due to their apatite-mineralization ability^2,3,7–12^. Different types of synthetic HydroxyApatite (HAp) are frequently used as coatings due to high biocompatibility and similarities with human bone constituents^22–31^. Several types of synthetic apatites are now commercially manufactured, being available for biomedical applications such as bone repair, augmentation and substitution and also for coatings on dental and orthopaedic implants^32,33^. Commercially available HAp coatings are predominantly produced by plasma-spray technique and have a layer thickness ranged from 50 to 200 μm. But such coatings have certain disadvantages such as poor adhesion, non-uniformity in coating density and poor control over the resulting HAp crystalline structure^34–38^. Additional problems arise with dense porous and lattice structures, as outer structural elements often “shade” inner ones from effective coating. Nowadays a special attention has been directed towards alternative techniques, which enable more predictable and controllable HAp deposition and more appropriate resulting layer crystalline structure^33,39–41^. Electrodeposition techniques (ED) are regarded as one of the most suitable methods for coating of complex additively manufactured metallic scaffolds. ED is rather inexpensive and does not need complex equipment operating in extreme environment. It allows obtaining a highly crystalline structure of coatings with low residual stresses and is capable of coating porous and geometrically complex structures^27–31^. Moreover, ED provides good control of the resulting coating properties and surface structures through adjusting deposition parameters such as applied potential and current density, deposition time, electrolyte temperature, ionic concentration and pH value^42–44^. Also a major role in the nucleation and growth mechanism of HAp is played by the crystallographic structure and orientation of the metal substrate. This mechanism is closely related to the *in vivo* biological mineralization process leading to the formation of teeth and bones, or the urinary calculi^45^. One of the possibilities to “tune” the crystalline phase structure of the as-manufactured metallic components is by additional heat and pressure exposure^46^, combined with machining or other types of mechanical treatment. Each additional post-processing step adds to the cost of the final product, and some of the desired types of post-processing are very hard to perform (e.g. with the structures having porous and lattice elements). Thus, industrially viable methods achieving coatings with desirable structure directly on as-manufactured surfaces of additively manufactured implants with complex structure are quite desirable.

It should be noted that “biomineralization” is a process which involves the *in vivo* formation of apatite structure in the extracellular space of the collagen and depends on various factors such as growth stage (e.g. development, fracture healing), region, age, etc.^47^. The bone nucleation is connected with interaction between the anionic proteins and the type I collagen, providing the negatively charged groups sufficient for HAp nucleation. After bone formation, the matrix starts to fast mineralize, being known as primary mineralization process which takes place in maximum 13 days. After a few days, the matrix will be mineralized up to 70%. The secondary mineralization process will start in the following several years, leading to an increase of 30% in mineral content^47^. Based on this observation, of primary importance in biological mineralization is understanding the formation of teeth and bones, as well as in pathological processes such as the development of urinary calculi^45^. Thus proving good potential of the coatings is not enough, but it should be complemented with the tests showing its further biomineralization ability.

The aim of the present study is to investigate the formation and properties of HAp coatings prepared by electrodeposition techniques (ED) on planar additively manufactured Titanium-Aluminium-Vanadium alloy (Ti64) metallic structures. The coatings were prepared in two different electrolyte solutions (acidic with pH = 5 and nearly neutral with pH = 6), to analyse the effect of pH on the characteristics of the resulting HAp coating. The investigation of microchemical, microstructural, morphological and electrochemical performance of the resulting HAp coatings was carried out. Special attention was paid to the experimental investigation of the ability for *in vitro* biomineralization of the coatings after 1, 3, 7, 14 and 21 days of immersion in SBF (pH = 7.4) at human body temperature (37 ± 0.5 °C), and to preliminary testing coated and uncoated samples *in vivo*.

## Results

### Microchemical and microstructural analysis of the uncoated Ti64 alloy substrate

**Figure 1:**
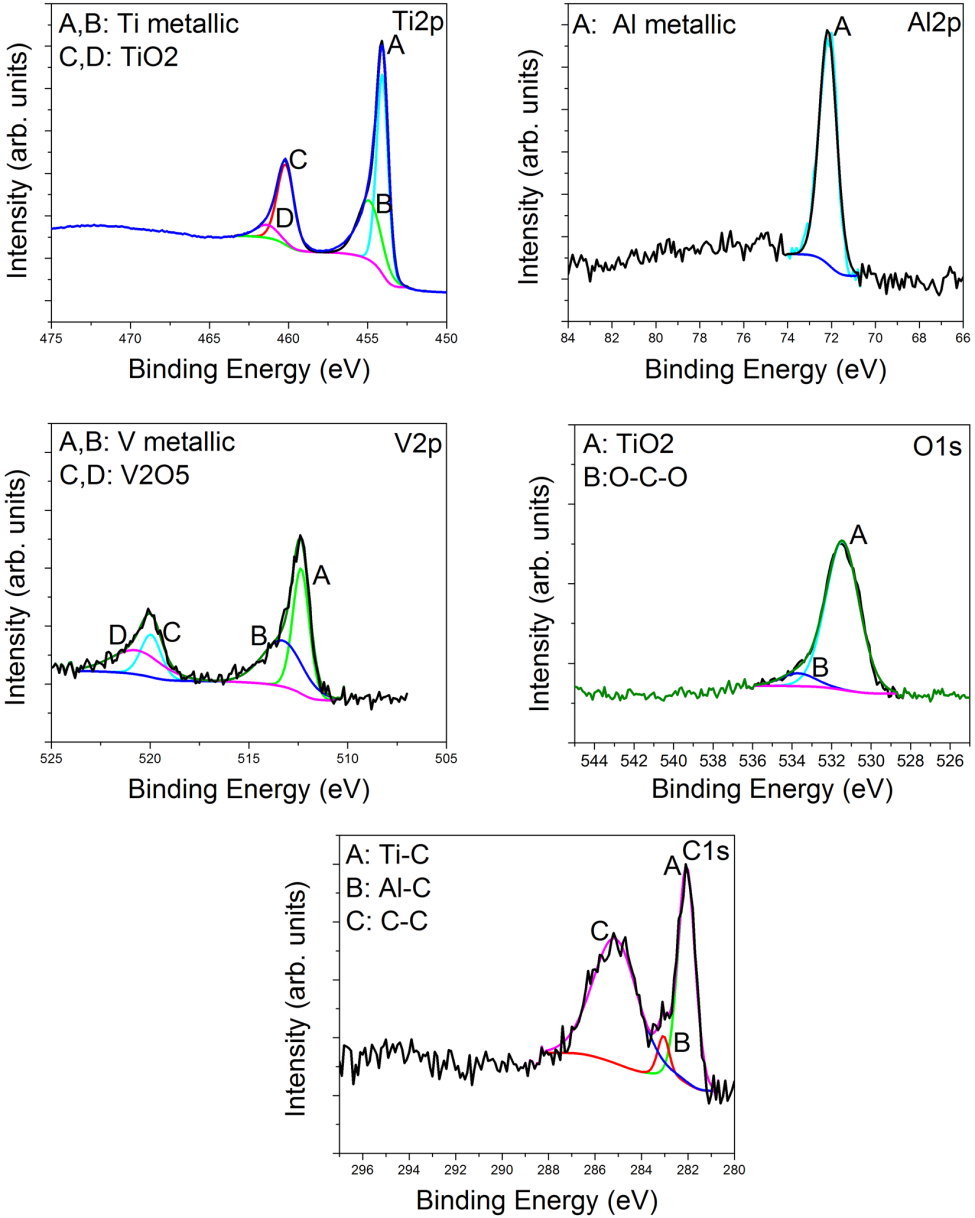
Ti2p, Al2p, V2p, O1s and C1s spectra of the uncoated porous Ti64 substrate prepared by the method of electron beam melting (EBM).

The XPS spectrum of the uncoated porous Ti64 alloy is presented in Fig. 1. If we take into account the XPS Ti2p peak, two different types of bonds can be found: Ti metallic and TiO_2_ at 457.1 eV and 460.2 eV respectively. The area of Ti metallic is found to be of about 69.5%, while for TiO_2_ is of 30.5%, indicating the formation of low oxide content. Based on the Al2p spectrum, only the Al metallic peak was found at 72.2 eV. According to the V2p peak, the V metallic and V_2_O_5_ oxide were found at 512.4 eV and 519.9 eV. The percent of V_2_O_5_ is approximately 34.7%, while V metallic is 65.3%. If we consider the XPS O1s peak, we can see the formation of Ti oxide (531.5 eV) and some O-C-O bonds (533.7 eV). From the C1s peak, three types of bonds were revealed: Ti-C (282.1 eV), Al-C (283.1 eV) and C-C bonds (285.4 eV). To summarize, the XPS experiments showed that the porous Ti64 sample produced by the method of electron beam melting (EBM) consisted of a mixture of some oxides and metals such as Ti, Al and V.

**Figure 2:**
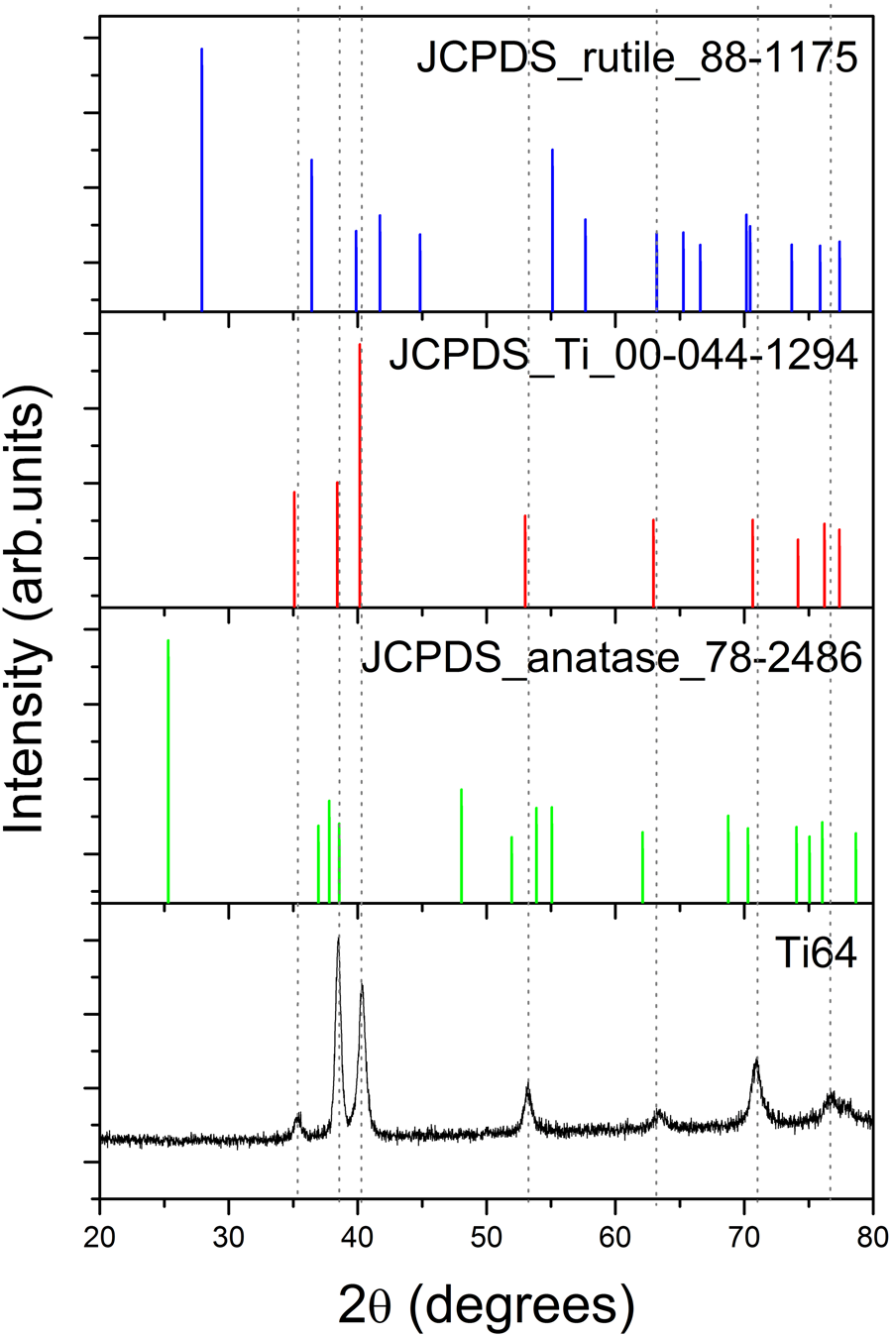
XRD diffraction pattern of the uncoated porous Ti64 substrate prepared by the method of electron beam melting (EBM) and of standard Ti-α pattern (JCPDS no. 044–1294), of Ti rutile phase (JCPDS no. 88–1175) and Ti anatase phase (JCPDS no. 78–2486).

X-ray diffraction pattern (XRD) of the uncoated porous Ti64 substrate is showed in Fig. 2. In this figure are also presented the corresponding spectrums of standard Ti-α pattern (JCPDS no. 044–1294), Ti rutile phase (JCPDS no. 88–1175) and Ti anatase phase (JCPDS no. 78–2486). One may observe that all peaks of our porous Ti64 alloy are closer to those of Ti-α pattern. Moreover, the peaks found at 38.4°, 53.3°, 70.9° and 76.7° can be also attributed to Ti anatase, while those positioned at 40.3°, 63.4° or 70.8° to the Ti rutile.

The XRD and XPS results are in good agreement, indicating that the porous Ti64 sample produced by electron beam melting (EBM) method comprised a mixture of some oxides and metals.

### Morphology and surface roughness of the coatings and Ti64 alloy substrate

**Figure 3:**
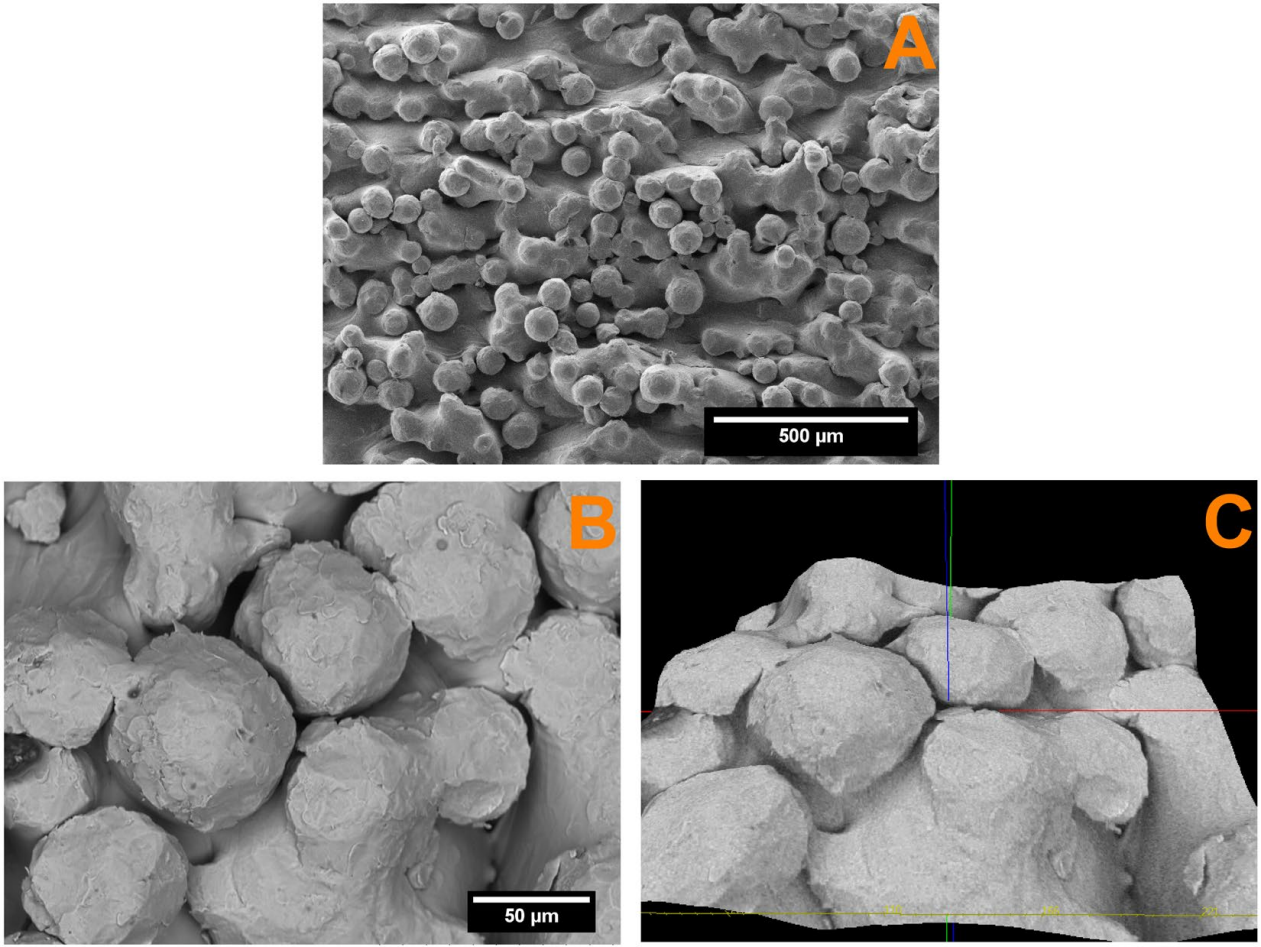
SEM images of the substrate surface (**A**) and SEM-3D images used for assessing the roughness of substrate surface (**B**,**C**).

The morphology and the substrate roughness has been assessed using SEM 3D images (Fig. 3). In order to determine the roughness of the substrate 20 profile lines were analysed on the Ti64 surface. The average roughness (Ra) value of the investigated area was 12.65 ± 0.83 µm.

From Fig. 3, it can be observed that the surface morphology is textured. Because in the powder-bed process (EBM) molten material on the component surface is surrounded by the ‘free’ powder some of the powder grains are only partially melted into the surface generating a characteristic surface roughness profile. Due to the specific processing conditions it is hardly possible to control the micro-roughness of the component surfaces below the grain dimensions in the powder-bed additive manufacturing. So additional nano-structuring of the surfaces together with biofunctionalization is often employed. Although several methods are available for metallic substrate biofunctionalization, electrochemical deposition seems to be more fitted for covering a substrate with micro-roughness as aforementioned, due to the fact that permits the electrocrystallization of HAp even in the hard to reach areas.

### Microchemical and microstructural analysis of the coatings

**Figure 4:**
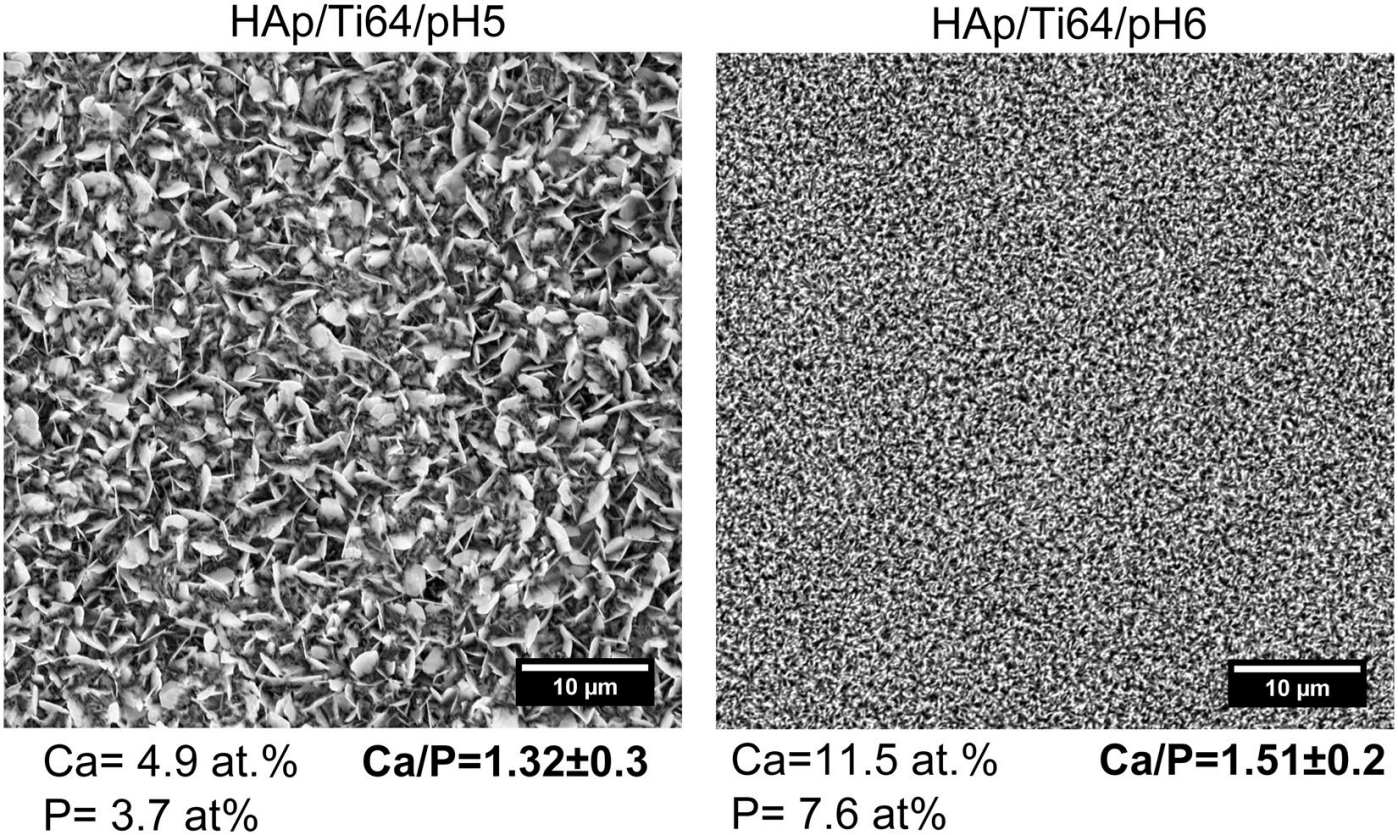
SEM images of the HAp/Ti64/pH5 and HAp/Ti64/pH6 coatings (×5000) and results of the ESD analysis of the surface.

Figure 4 presents SEM (scanning electron microscopy) images of the HAp coatings on the disc samples prepared using electro deposition in the electrolytes with pH = 5 and 6, and respective atomic contents of Ca and Ca/P ratios. Note that the surface of Ti64 substrate is fully covered with HAp independent of the pH of the electrolyte. On the other hand, pH influences the morphology of the coatings. At pH = 5 plate-like crystals, nano-flakes and nano-walls are seen on the surface indicating the abundance of pores. Coatings deposited in less acidic electrolyte (pH = 6) contain large number of needle-like crystals of dimensions at least one order of magnitude smaller than previously. It can also be noted that these needles/nano-flake crystals stick out from the substrate along normal to the surface.

Based on the EDS and SEM results, it can be concluded that the values of Ca/P ratio of the electrochemically deposited HAp coatings are close to those of stoichiometric HAp (Ca/P = 1.67) in both cases. The coatings prepared at pH = 6 exhibited the Ca/P ratio closer to the value of stoichiometric HAp.

**Figure 5:**
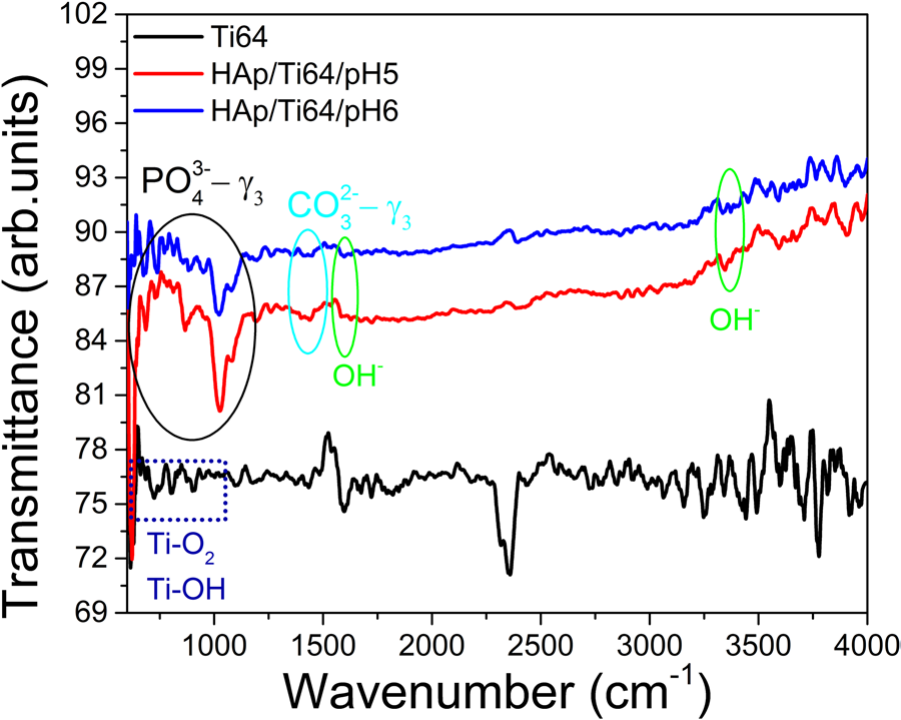
The FT-IR spectra of the Ti64 substrate and coated sample (HAp/Ti64/pH5 and HAp/Ti64/pH6 coatings).

Figure 5 presents the FTIR (Fourier transform infrared spectrophotometry) spectra of HAp electrodeposited in the electrolytes with pH = 5 and 6 and substrate. The coatings spectra are similar, and the bands specific to HAp can be identified in both cases. The bands between 594 and 1100 cm^−1^ are attributed to the ${{\rm{PO}}}_{4}^{3-}$ −ν_3_ vibrations; the peaks ranged from 1300 to 1500 cm^−1^ are indicative of the ${{\rm{CO}}}_{3}^{2-}$ −ν_3_ vibrations; the bands corresponding to the absorption peaks of OH^−^ groups are observed at 3450 cm^−1^ and 1650 cm^−1^. Similarity of the FTIR spectra for the coatings prepared in the electrolytes with different pH values indicates that in the chosen range acidity of the electrolyte has no significant impact on the ED coating composition. The Ti64 characteristic oxides and metal-OH bonds on alloys surface, appear at 1100–450 cm^−1^. Presence of the “noise” in spectra can be attributed to a significant roughness of the surfaces under study and did not make possible the identification of the characteristic peaks for oxides and metal-OH bonds.

**Figure 6:**
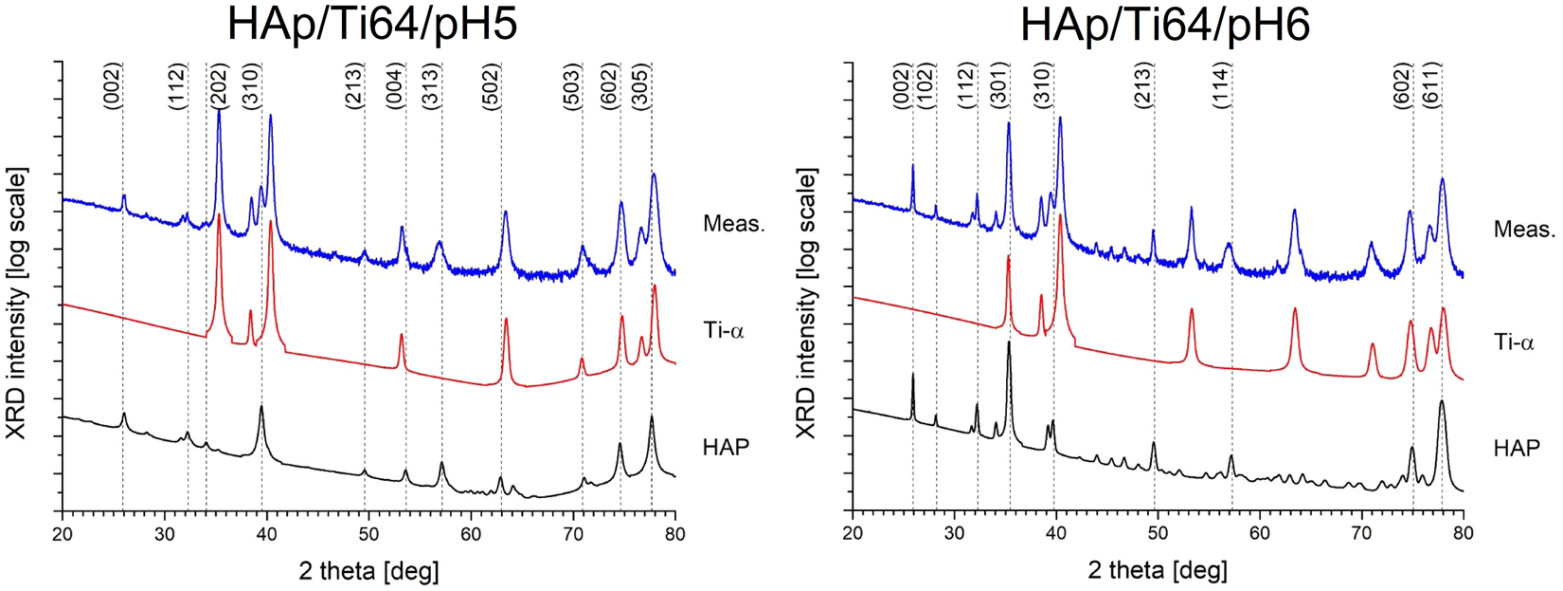
Semi-log plots of XRD spectra of the samples under study with numerically extracted sub-patterns coming from the component materials (Ti-α and stoichiometric HAp): HAp/Ti64/pH5 (top), HAp/Ti64/pH6 (bottom). HAp structure is labelled with respective (hkl) indices.

**Figure 7:**
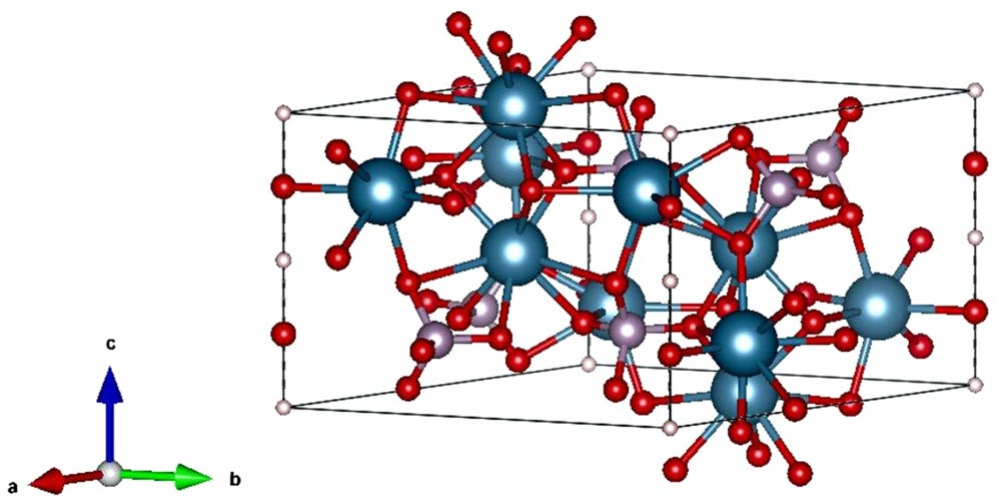
Model of a primitive cell of a HAp crystal plotted using VESTA software application^49^.

Figure 6 presents X-ray diffraction patterns (XRD) of the coated (HAp/Ti64/pH5 and HAp/Ti64/pH6) and uncoated (Ti64) disc samples. Corresponding spectrum of the uncoated sample is dominated by the Ti-α pattern. Table 1 summarizes structural properties of the samples considering various phase components determined from XRD spectra using Rietveld refinement method and Williamson-Hall plot^48^. Schematic plot of a unit cell of HAp crystal plotted using the VESTA software^49^ application is shown in Fig. 7.

**Table 1:** Structural properties of the samples under study derived from XRD spectra using the Rietveld algorithm: a_0_, c_0_ – lattice constants along crystallographic axes, Phase cont. – Volumetric phase contribution, CDD size – the size of coherently-diffracting domains, ε – relative structural microstrain values.

Sample	Ti(α)					HAp					
	a_0_ (Å)	c_0_ (Å)	Phase cont. (vol. %)	CDD size (Å)	ε (%)	a_0_ (Å)	c_0_ (Å)	Phase cont. (vol. %)	CDD size (Å)	ε (%)	Ca/P
Ti-α	2.950	4.680	—	—	—	9.400	6.850	—	—	—	1.67
HAp/Ti64/pH5	2.928	4.677	12	410	0.08	9.485	6.828	88	200	−0.05	1.32
HAp/Ti64/pH6	2.930	4.667	29	2100	0.29	9.428	6.838	71	1400	0.26	1.51

It turned out that the coatings deposited in more acidic solution are thicker due to higher phase contribution of the HAp relative to the underlying Ti-α substrate (88 and 12% respectively), as compared to the one deposited in more neutral electrolyte (71 and 29%). On the other hand, the latter coatings contain larger mosaic areas strongly responsible for coherent diffraction of incident X-rays. The size of coherent diffraction domains (CDDs) in this sample is 2100 and 1400 Å for Ti-α and HAp, respectively, which is 5–7 times higher to those deposited at pH = 5 (410 and 200 Å, respectively). However, the size of the CDD also influences relative microstructural stresses generated within the component sub-structures: larger domains correspond to higher microstrains. Note that the coatings deposited in more acidic solution appear to be almost void of internal stress (0.08 and −0.05 percent in Ti-α and HAp, respectively), whereas those at pH = 6 are more distorted (0.29 and 0.26 percent in Ti-α and HAp, respectively). At the same time, lower mosaic area sizes correspond to larger level of low-angle defects and inter-grain boundaries responsible for previously mentioned stress-relief behaviour of the structures under study. In general, the larger grains in the less stoichiometric material contain much smaller although less distorted sub-domains.

### Morphology and surface roughness of the coatings

The composition and morphology of the resulting HAp coatings depends on the reaction media pH, temperature, and solution composition^50^. The mechanism of electrochemical deposition (ED) of HAp coating in a Ca-P electrolyte system has also been discussed in some studies^50,51^.

Under cathodic polarization, several electrochemical and chemical reactions are potentially involved in the electrochemical deposition mechanism on Ti and its alloys as follows:

1 $${O}_{2}+2\,{H}_{2}O+4\,{e}^{-}\to 4\,{(OH)}_{ads}^{-}$$

2 $$2\,{H}^{+}+2\,{e}^{-}\to {H}_{2}$$

3 $$2\,{H}_{2}O+2\,{e}^{-}\to {H}_{2}+2{(OH)}_{ads}^{-}$$

4 $$2\,Ti{O}_{2}+2\,{H}_{2}O+{e}^{-}\to Ti{(OH)}_{3}+{(OH)}_{ads}^{-}$$

5 $${H}_{2}P{O}_{4}^{-}+{H}_{2}O+2\,{e}^{-}\to {H}_{2}P{O}_{3}^{-}+2\,{(OH)}_{ads}^{-}$$

6 $${H}_{2}P{O}_{4}^{-}+{e}^{-}\to {H}_{2}P{O}_{4}^{2-}+\frac{1}{2}{H}_{2}$$

7 $${H}_{2}P{O}_{4}^{-}+2\,{e}^{-}\to P{O}_{4}^{3-}+{H}_{2}$$

8 $${H}_{2}P{O}_{4}^{-}+{e}^{-}\to P{O}_{4}^{3-}+\frac{1}{2}\,{H}_{2}$$

9 $$5\,(C{a}^{2+})+3\,P{O}_{4}^{3-}+{(OH)}^{-}\to C{a}_{5}{(P{O}_{4})}_{3}OH$$

The principle of the electrochemical-assisted HAp deposition on metallic substrate under cathodic polarization is based on the pH-dependent solubility of calcium phosphate. This method increases the pH at the interface between the substrate and the electrolyte (reaction 1 through 5) due to electron incorporation, forming OH^−^ ions and H_2_ through water reduction^51^. The increase in concentration of hydroxyl ions results in increased concentration of phosphate ions, which are mandatory for the deposition of HAp (reaction 9). Even when significant concentrations of phosphate ions are produced by reduction of hydrogen phosphate ions and dihydrogen phosphate ions (reactions 8 and 9), one should consider the fast kinetics of the phosphate ions recombination with hydrogen ions. This implies that the concentration of calcium ions in solution must be higher compared to the concentration of hydrogen ions, allowing the precipitation of CaP in solution by reaching the solubility limit.

The sudden increase in pH induces crystal nucleation and initiates crystal growth of the desired CaP phase directly on the substrate surface. HAp is preferentially deposited when the pH of the electrolyte adjacent to cathode surface is higher than around 4.5 because the solubility of HAp is lower than those of other CaP’s such as dicalcium phosphate dehydrate (DCPD) and octacalcium phosphate (OCP)^52^, but by using an acidic CaP solution as the electrolyte, DCPD or OCP is likely to be deposited.

Classical nucleation theory, predicts that crystal formation from a supersaturated solution of its constituent ions requires local fluctuations in concentrations of the interacting ions.

At a critical concentration of those ions their favourable interactions stabilize the ion cluster so that the surface free energy gain by dissociation of the ions is less than the free energy gain by further addition of ions to the crystalline phase.

**Figure 8:**
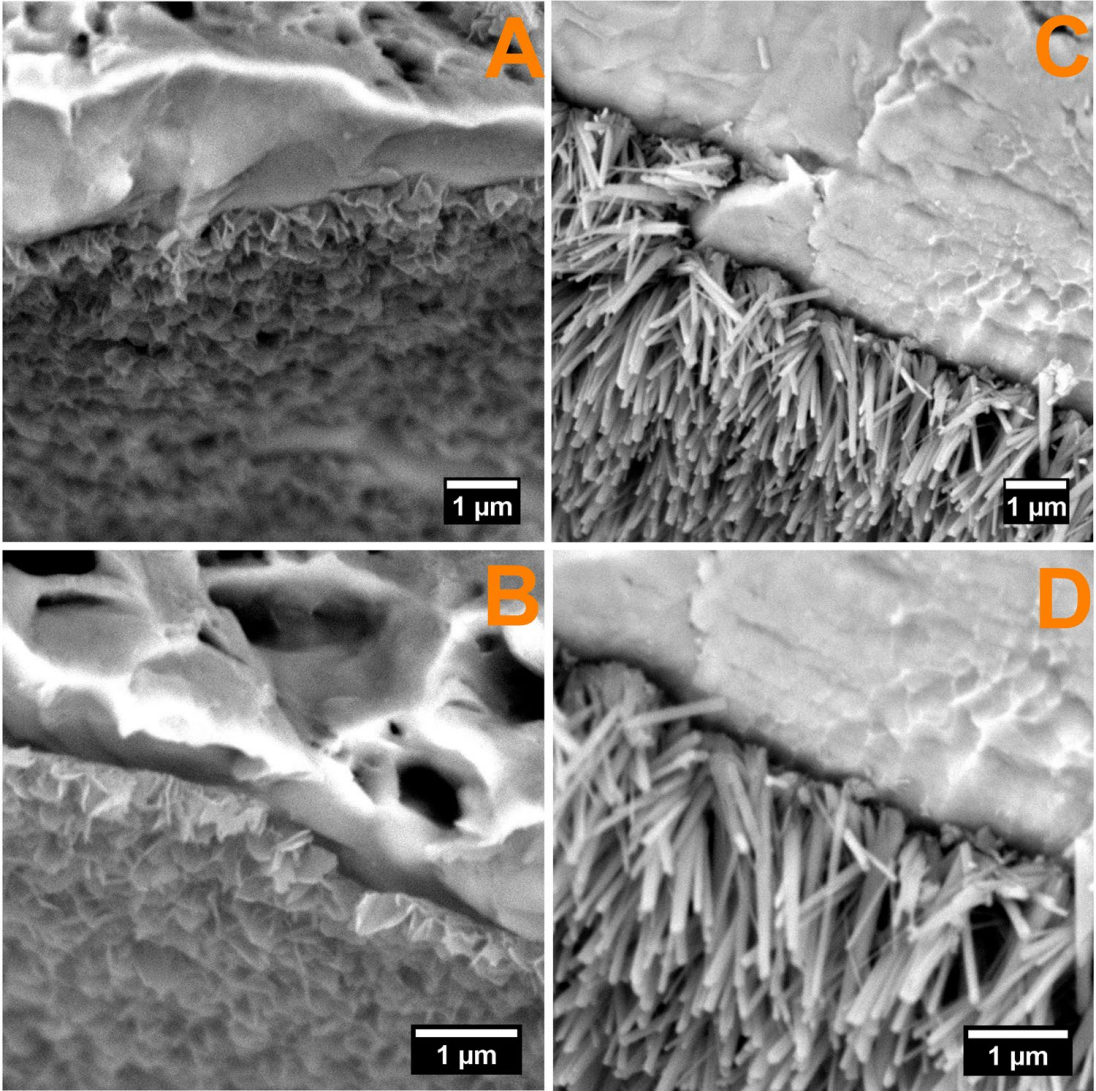
Cross section SEM images for HAp/Ti64/pH5 (**A**,**B**) and HAp/Ti64/pH6 (**C**,**D**).

Considering all the aforementioned it can be assumed that as the pH of the electrolyte increases, the kinetics of electrochemical reactions decreases, thus at pH = 5 the reactions that are leading to formation of hydrogen gasses is more energetic compared to pH = 6, and hydrogen released volume being higher thus favouring the growth of HAp with plate like morphology (Fig. 8), while at pH = 6 the hydrogen volume is lower, favouring the needle like morphology (Fig. 8). Figure 8 presents the SEM cross section images of the coated samples. As it can be seen both coatings are presenting the same preferred orientation, perpendicular on the substrate, regardless the topography and/or roughness of the substrate.

The thicknesses of the coatings were measured using cross-section SEM images and the results are 455 ± 27 nm and 2,270 ± 97 nm for HAp obtained at pH = 5 and HAp obtained at pH = 6 respectively.

**Figure 9:**
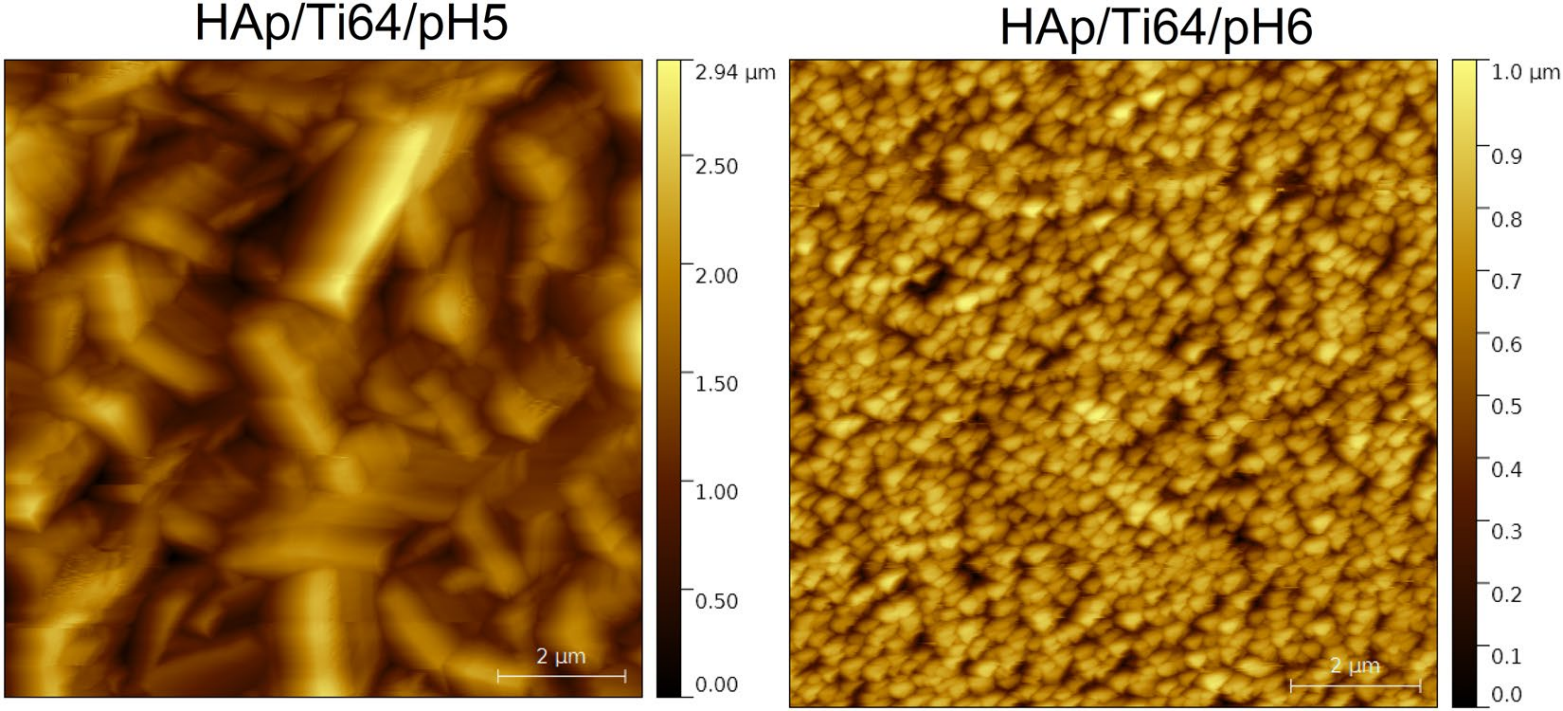
AFM images of the HAp samples (10 × 10 µm^2^) deposited at various acidity of the electrolyte: HAp/Ti64/pH5 and HAp/Ti64/pH6.

Figure 9 presents AFM (Atomic Force Microscopy) images of the coated disc samples taken with 512 pts resolution and 10 µm scan length. HAp grains substantially differ in their size and shape depending on the pH value of the electrolyte. Deposition in more acidic liquid results in larger grains with sharper edges constituting well-developed external shapes of the crystals. Alternatively, deposition in more neutral electrolyte produces structures composed of much smaller, regular grains. Grain analysis, using Otsu algorithm^53^, revealed that average grain size for the HAp/Ti64/pH5 sample exceeds 500 nm, whereas for the HAp/Ti64/pH6 sample approaches only 250 nm.

**Figure 10:**
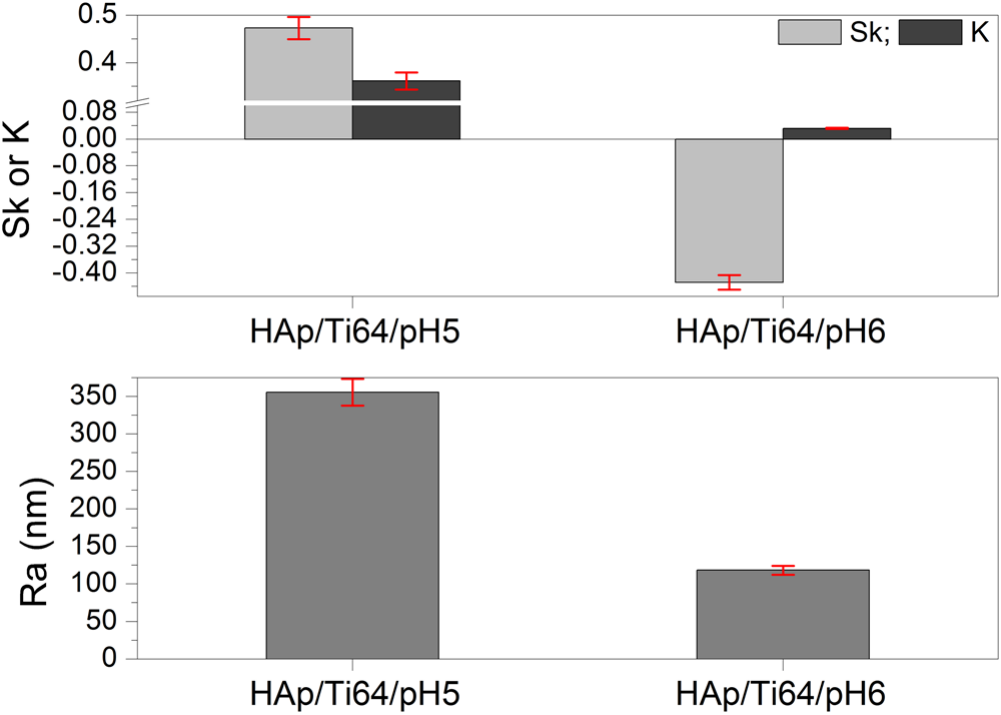
Roughness parameters of the coated surfaces by AFM measurements, including the bars indicating error margins.

The main roughness parameters, calculated from the AFM images, are presented in Fig. 10. Note that average surface roughness (R_a_) of the coatings is decreasing with increasing the pH of used electrolyte. The R_a_ value of the coatings prepared at pH = 6 is about 3 times lower than that for the coating prepared at pH = 5.

Both coatings have a kurtosis less than 0.4, the coating prepared at pH = 6 exhibited a kurtosis value close to zero (0.032), indicating that this coating is uniformly grown. The kurtosis parameter decreases from 0.36 to 0.03, indicating a transition from a sharp with large tails one to broader one and shorter tails.

Explanations of the meaning for the kurtosis and skewness parameters can be used to better understand their meaning. Skewness and kurtosis of a flat surface are zero. Negative skewness indicates that a surface of material consists of many peaks with heights close to the mean height value. Positive skewness indicates a surface with a wider range of peak heights that are predominantly higher than the mean “landscape level”. If the kurtosis value deviates from 0 it indicates that the roughness features are not normally distributed. Negative kurtosis means that some deep grooves are present on the surface. It is reported that the surfaces with few high peaks and low valleys typically have s the kurtosis less than 3^54^. In our case, Skewness and kurtosis are two roughness parameters which can indicate the differences in the corrosion performance of a material, and could be used as guide for estimation of the corrosion resistance^55^. Evgeny *et al*. found that increasing positive skewness is an advantage for having good corrosion resistance, while surfaces with negative skewness mainly exhibit pitting corrosion^56^. Basing on this suggestion one may conclude that coatings deposited in the electrolyte with pH = 5 having higher positive skewness values should probably have better corrosion resistance. Also, if the low kurtosis values indicating that a surface is composed of high peaks with small deep valleys that should provide low contact area with corrosive solution, should have a better corrosion resistance^57^. The coating prepared at pH = 6 have low kurtosis, and these samples also should have good corrosion resistance. Thus it is predicted that both coatings should have rather good corrosion resistance, but basing on the roughness parameters alone it is hardly possible to predict which samples will be better, and direct experiments should be conducted.

### Electrochemical studies

**Figure 11:**
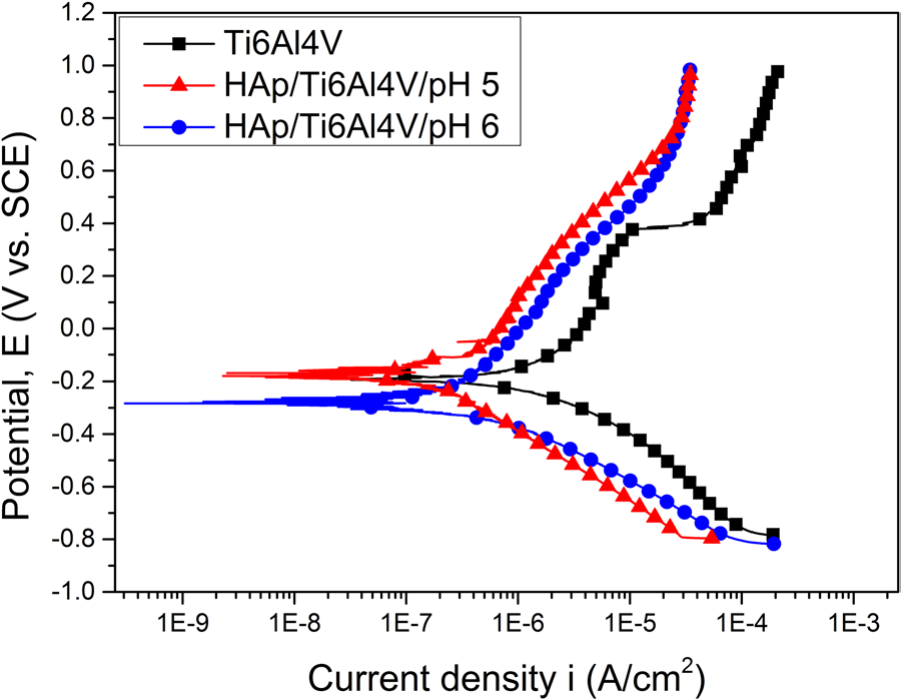
Potentiodynamic curves of the uncoated sample (Ti64) and samples coated in the electrolytes with different acidity (HAp/Ti64/pH5 and HAp/Ti64/pH6).

For the corrosion resistance measurements uncoated Ti64 discs were used as a control sample. According to Baboian^58^ and Mansfeld^59^, a material is resistant to the corrosive attack of a media when all of the following are met: a more electropositive corrosion potential (E_corr_), a higher polarization resistance (R_p_) and low corrosion current density (i_corr_). Figure 11 presents the potentiodynamic curves for the coated and uncoated samples. The electrochemical parameters extracted from the experimental data using the Tafel extrapolation^58^ are presented in Table 2.

**Table 2:** Electrochemical parameters extracted with the help of Tafel extrapolation: E_corr_ – corrosion potential; i_corr_ – current density; R_p_ – polarization resistance; P_e_ – coatings protective efficiency; P – porosity.

Sample	E_corr_ (mV)	i_corr_ (µA/cm^2^)	R_p_ (kΩ∙cm^2^)	P_e_ (%)	P (%)
Ti64	−188.96	1295.00	48.06	—	—
HAp/Ti64/pH5	−177.39	143.11	335.18	88.9	0.14
HAp/Ti64/pH6	−289.00	91.15	397.07	92.9	0.09

The corrosion measurements in SBF solution allow concluding that both samples have a good corrosion resistance, which is better than for the uncoated Ti64 substrate. It supports the prediction gained from the roughness measurements. It also means that despite the presence of pores deposited HAp coatings provide good substrate surface protection from the corrosive media.

As it can be seen in Table 2, better results were obtained for the samples with coating deposited in more neutral electrolyte (HAp/Ti64/pH6), which has smaller corrosion current density (i_corr_) and the higher polarization resistance (R_p_). From the point of view of resistance to the corrosive attack, both samples recorded very good values, observing however that deposits at pH = 6 have a higher P_e_ value of ~93%, compared with throne for the coatings deposited at pH = 5 (~89%), indicating that the HAp/Ti64/pH6 samples have a higher resistance to corrosion in SBF solution overall. The obtained results are in strong correlation with the findings by N. Eliaz^60^ reporting that the corrosive behaviour of HAp coatings prepared by electrochemical deposition at pH = 6 were more resistant to corrosive attack.

### *In vitro* biomineralization ability

**Figure 12:**
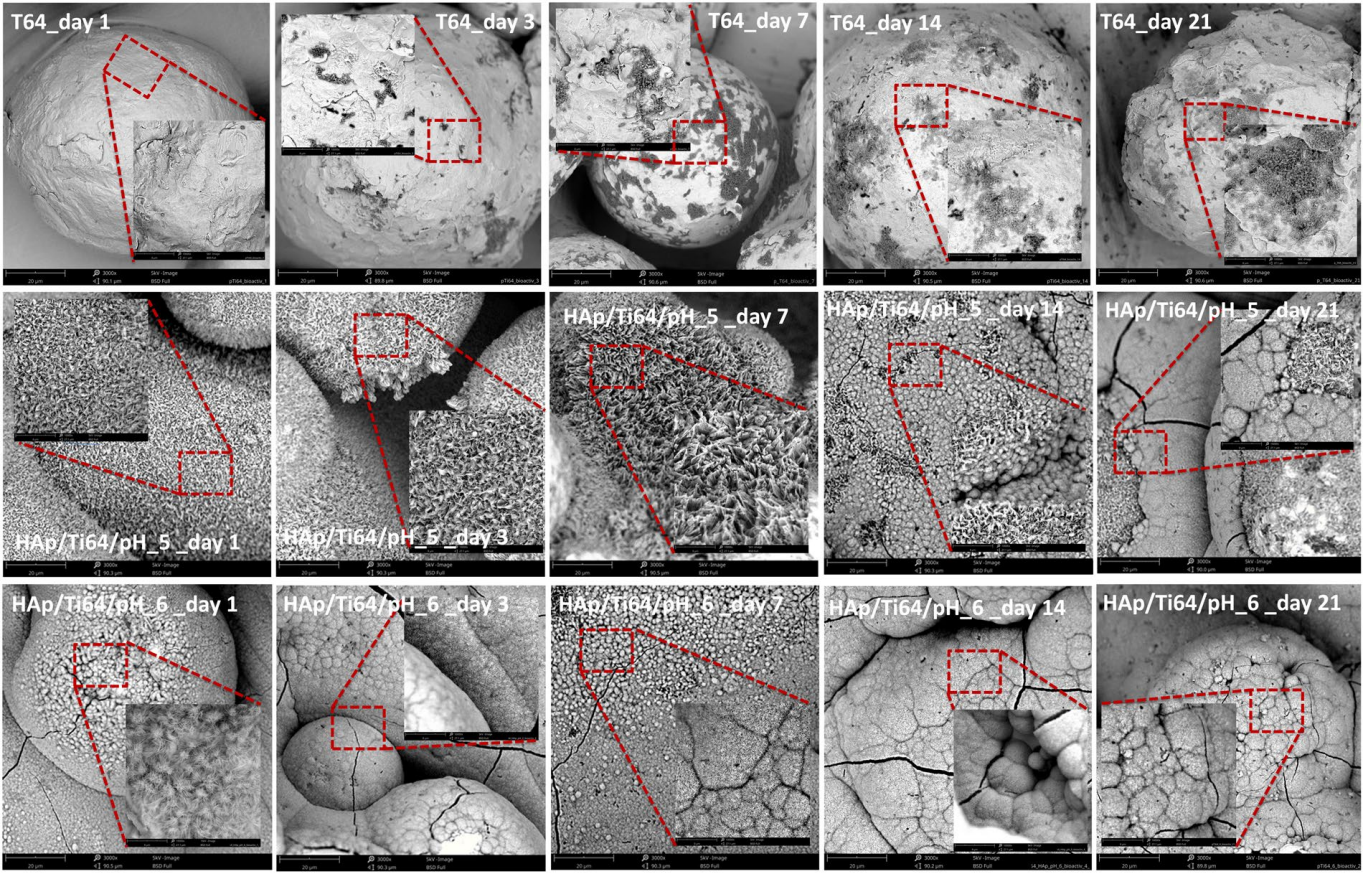
SEM images of the samples’ morphology after bioactivity tests. Main image: magnification ×3000, inserts- × 10000.

**Figure 13:**
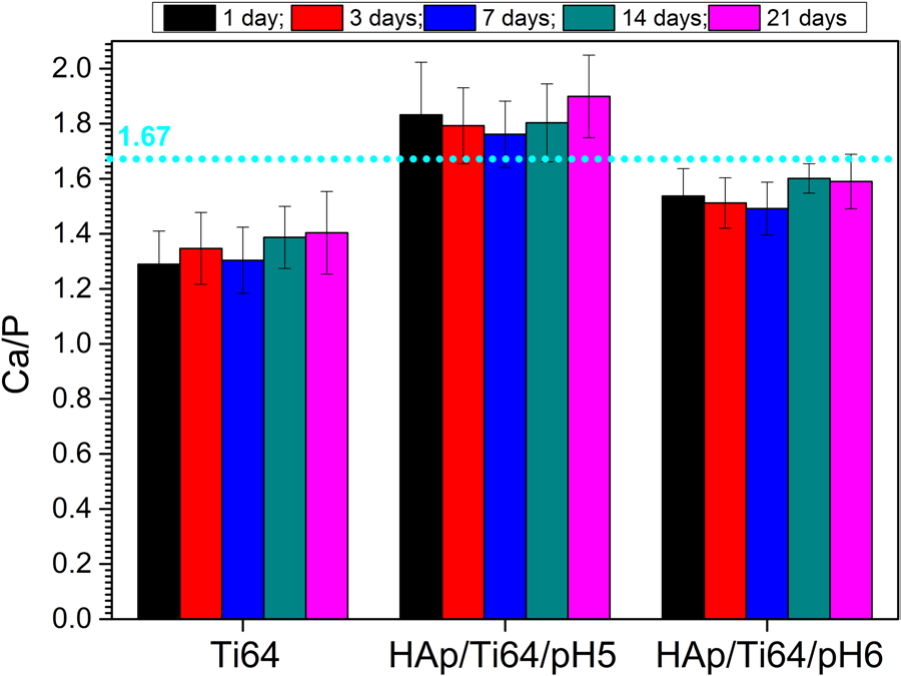
The Ca/P ratio of newly formed apatite layer on surfaces of samples after bioactivity tests.

*In vitro* bioactivity assessment was performed using coating mineralization ability in the simulated body fluid. Figure 12 presents the SEM images of uncoated and coated samples after bioactivity tests. On the surfaces exposed to the SBF media, apatite structures were formed, independent of the pH of the electrolyte used for coating. Another notable result is given by the fact that the newly apatite structures were formed on the experimental samples independent on the SBF exposure duration. After 21 days of immersion, coatings on all samples were predominantly covered in hemispherical precipitates with some cracks. HAp/Ti64/pH6 samples started to develop hemispherical features much earlier than HAp/Ti64/pH5 ones, demonstrating an accelerated biomineralization process. After only 3 days of immersion semispherical features were covering the entire HAp/Ti64/pH6 sample surface, while the surface of HAp/Ti64/pH5 samples was not completely coated with these features after 21 days of immersion. According to the EDS analysis Ca, P and O are present in the newly formed structures. In the case of uncoated Ti64 substrate, even after 21 days of immersion these new surface features were not developing effectively, indicating rather poor ability for *in vitro* biomineralization. For the same SBF immersion time, the coatings prepared at pH = 6 reveal more new apatite formations over entire surfaces, indicating high bioactive abilities. Figure 13 presents Ca/P ratios calculated basing on the EDS results for coated and uncoated samples for 1, 3, 7, 14, and 21 of SBF exposure. For the uncoated Ti64 samples Ca/P ratio slightly increased with the SBF exposure time, still being below the stoichiometric value of apatite. Coated HAp/Ti64/pH5 samples have Ca/P ratio some higher than that of stoichiometric HAp (1.67) for all SBF exposure times. Coated HAp/Ti64/pH6 samples have Ca/P ratio some lower than that of stoichiometric HAp coming rather close to it after 7 days of SBF exposure. These results show that coatings prepared at both pH = 5 and pH = 6 favour the formation of new apatite structure with the Ca/P ratio close to that of stoichiometric HAp when exposed to SBF.

**Figure 14:**
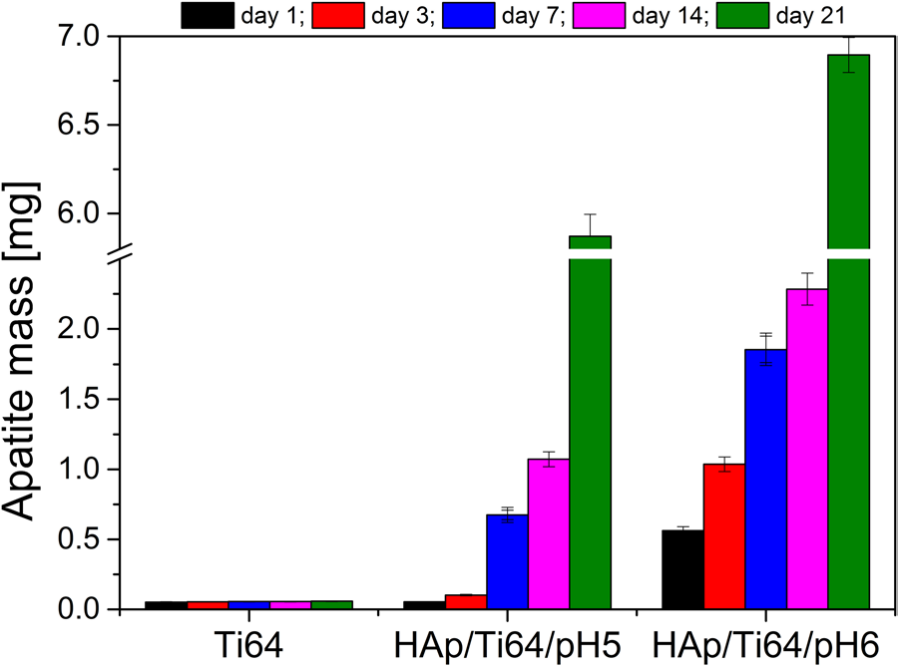
*In vitro* bioactivity evolution in term of apatite mass gain for Ti64, HAp/Ti64/pH5 and HAp/Ti64/pH6 samples.

Figure 14 shows the dynamics of the mass increase for the samples exposed to SBF. While uncoated Ti64 samples were not showing any significant additional mass, both coated samples acquired additional HAp quite rapidly. The best biomineralization ability was found in the case of HAp/Ti64/pH6 (coatings prepared at pH = 6), which after 21 days of SBF exposure have registered an additional apatite mass approximately three times higher than that at 14 days (almost 7 mg in total). The HAp/Ti64/pH5 coatings have gained about 6 mg of apatite after 21 days of SBF exposure, which is quite close to the additional mass gained by HAp/Ti64/pH6 samples. Corresponding mass gain per surface unit of the coated samples is 5.5 and 4.71 mg/cm^2^ after 21 days of exposure to SBF.

Li *et al*.^61^ has suggested that the apatite is generated by the formation of electrical double layer within the interface of immersed specimen and the synthetic body fluid (SBF). The bioactive coatings surface obtains negative charges during its degradation and at the same time the ionic exchange in the SBF occurs. The attraction of calcium ions and the formation of electrical double layer within interface leads to supersaturation of electrical double layer solution with respect to the HAp and its deposition/formation on the surface. This cascade of events is being closely guided by the electrostatic interaction of the coated bioactive surface functional groups and the calcium and phosphate ions in the fluid^62^.

This indicates that the biomineralization of bone-like apatite on the HAp is a smart process which involves the electrostatic interaction of the HAp surface with the calcium and the phosphate ions which leads to a newly formed apatite layer.

In respect to the developed coatings, an important aspect which need to be considered is the surface to volume ratio that is higher for the needle like morphology (pH = 6) when compared to plate like morphology (pH = 5), favouring so the biomineralization process in SBF media. Nevertheless some other aspects should be considered as favouring factors among which a Ca/P ratio closer to the stoichiometric one (Ca/P = 1.67) and the higher crystallinity in the case of pH = 6 when compared to pH = 5.

### *In vivo* assay

**Figure 15:**
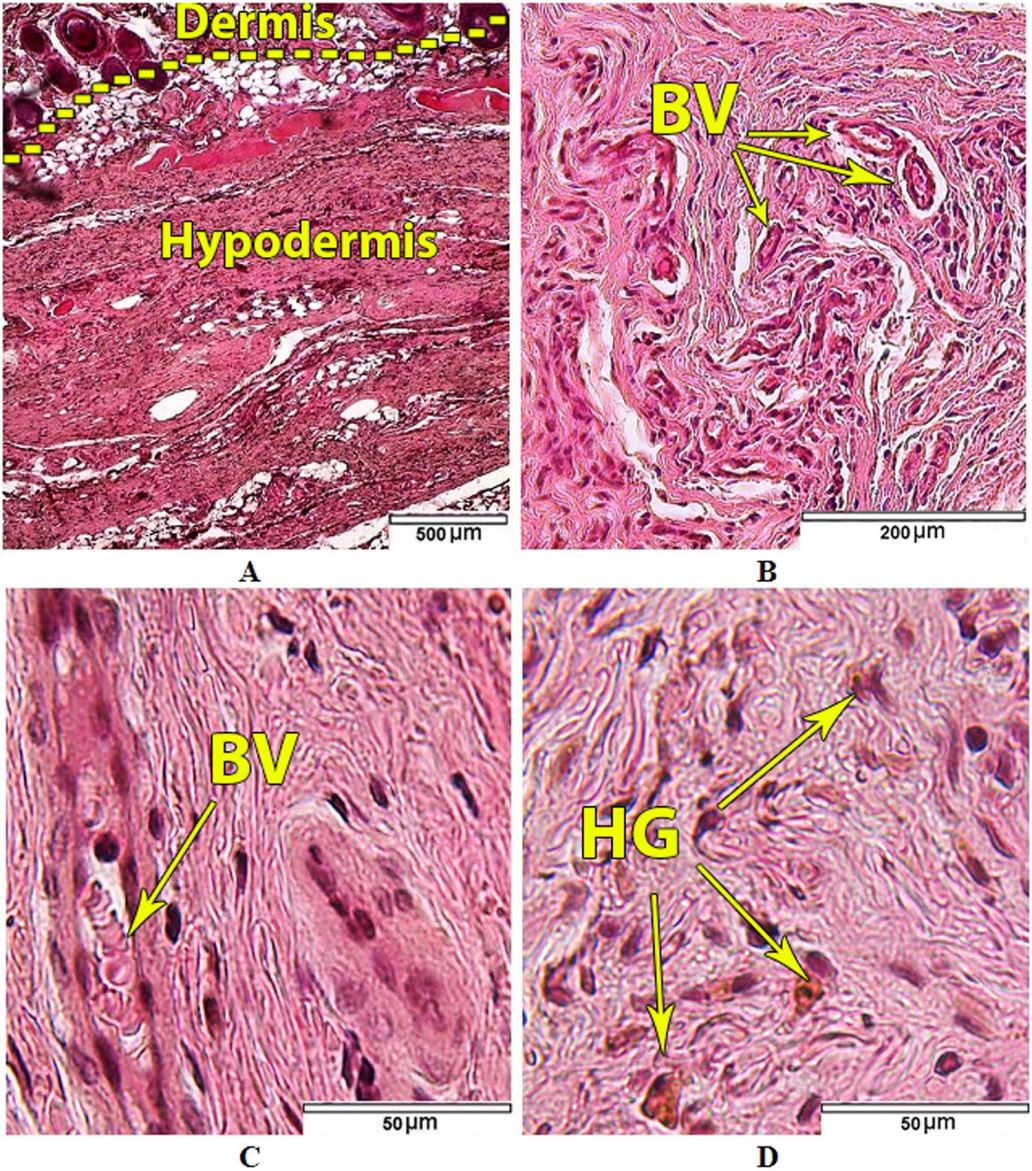
Tissues adjacent to the Ti64 control samples. (**A**) Dermis and hypodermis; (**B**) Dermal vessels; (**С**) Cellular populations and blood vessels (BV) of hypodermal connective tissue; (**D**) Free lying hemosiderin granules (HG) in hypodermal connective tissue. Hematoxylin and eosin staining.

The *in vivo* biological tests were performed only on uncoated samples (as a control) and substrate coated with HAp/Ti64/pH6 which showed the superior electrochemical and *in vitro* bioactivity results. Both types of samples were not subjected to the *in vitro* biomineralization in SBF. The morphology of the skin in the area adjacent to the upper surface of the control Ti64 samples corresponded to the it’s normal histological structure (Fig. 15A,B). Arterial as well as venous vessels of the dermis and hypoderm in perifocal zone were marked by moderate blood filling (Fig. 15B,С). Vascular permeability violations and the formation of soft tissue edemas in the animals of the control group were not observed. No signs of fresh haemorrhages were detected. But in the half of the control group animals single siderophages or those forming small clusters (from 2 to 4 cells), as well as free-laying granules of hemosiderin were found in the soft tissues of implantation area, in particular at the border of dermis and hypoderm (Fig. 15D).

Siderophages and hemosiderin in soft tissues displayed the presence of old, resorbable small haemorrhages. It was found that fibroblasts and fibrocytes were predominant in cellular populations of the hypodermal connective tissue adjacent to the control Ti64 samples (Table 3). Leukocyte infiltration of tissues adjacent to the control samples was not observed (Table 3). Only single lymphocytes and macrophages were visualized (Fig. 15C,D).

**Table 3:** Cellular populations composition (average number of cells) of hypodermal connective tissue adjacent to control and experimental samples of the Ti64 alloy (Lens 63x).

Group of animals Cell type	Control n = 10	Experimental n = 10
Fibroblasts	19 (15; 28)	23 (15; 32) р = 0.259684
Fibrocytes	16 (12; 23)	17 (12; 23) р = 0.539513
Macrophages	0 (0; 1)	0 (0; 1) р = 0.955351
Foreign-body giant cells	0 (0; 0)	0 (0; 0) р = 0.865877
Lymphocytes	0 (0; 0)	0 (0; 1) р = 0.714834
Neutrophils	0 (0; 0)	0 (0; 0) р = 0.997250
Eosinophils	0 (0; 0)	0 (0; 0) р = 0.997250

**Figure 16:**
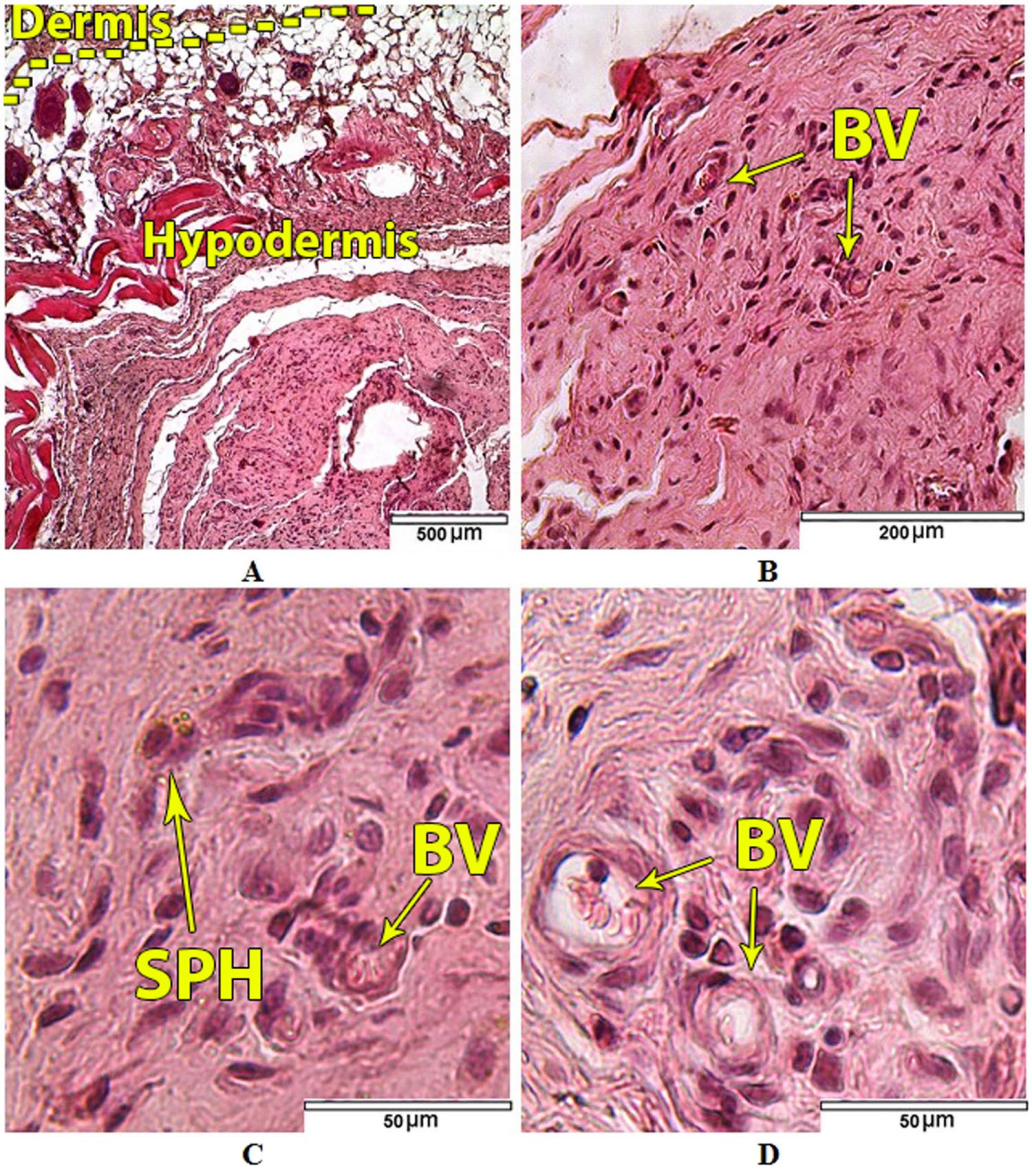
Tissues adjacent to the HAp/Ti64/pH6 experimental samples: (**A**) Dermis and hypodermis; (**B**) Blood vessels (BV) in hypodermal connective tissue; (**C**) Single siderophage in hypodermal connective tissue; (**D**) Cellular populations of hypodermal connective tissue. Hematoxylin and eosin staining.

In the histological specimens of the skin of the area adjacent to the experimental HAp/Ti64/pH6 samples no pathological changes were found (Fig. 16A,B).

Both arterial and venous vessels of the dermis and hypodermis in the sites adjacent to the experimental HAp/Ti64/pH6samples were characterized by moderate blood filling (Fig. 16B). No signs of fresh haemorrhages were found in any of the animals of that group. Single siderophages in the dermis and hypodermis were found in 6 animals of that group (Fig. 16C), reflecting old resorbable small-focal haemorrhages, which were probably caused by small vessels traumatization during sample implantation. Manifestations of vascular wall permeability disorders such as edema formation in animals of this group were absent. The composition of cellular populations of the hypodermal connective tissue in the sites adjacent to the experimental HAp/Ti64/pH6samples corresponded to the normal structure of а loose connective tissue. Fibrocytes and fibroblasts were the main cell types in hypodermal connective tissue (Table 3). Leukocyte infiltration of tissues adjacent to the experimental HAp/Ti64/pH6 samples was not revealed (Fig. 16C,D), only single leucocytes including macrophages and lymphocytes were detected. There were no statistically significant differences in the cells number of each population in the control and experimental groups (Table 3).

## Discussions

The responses of human body to implanted biomaterials involve many complex processes and in order to achieve successful implantation, it is very important to know more about the interaction between the biomaterials and human tissue before the insertion of biomaterials. The best way to find these is to analyse the biomaterials by *in vitro* and *in vivo* tests in environments close to the real one found in human body. Coating structure together with surface micro- and nano-scale topography significantly influence early stages of the implant bio-integration. In this study, we analysed the effect of pH modification on the morphology, *in vitro* corrosion behaviour, *in vitro* bioactivity and *in vivo* biocompatibility of HAp coatings prepared by ED on the additively manufactured Ti64 samples. The HAp coatings can be successfully prepared by means of electrochemical assisted deposition (ED). Such coatings are intensely used for biomedical implants, but ED coating may be problematic for the structures with complex geometry including lattices, and with porous surfaces. In this study, the properties of coatings prepared in two different electrolyte solutions (acidic with pH = 5 and nearly neutral with pH = 6) were compared.

The experimental results showed that the coatings deposited in the electrolyte with pH = 6 are denser with needle like crystals with just few plate-like elements of smaller dimensions. The coatings prepared in a solution with pH = 5 exhibited larger grains with sharper edges constituting well-developed external shapes of the crystals. Alternatively, the coatings obtained in the electrolyte with pH = 6 deposition revealed structures composed of much smaller, regular grains. These results are key features in resistance of corrosive attach of human solution as well in mediating of the cell attachments or biomineralization abilities. It is feasible that higher corrosion performance of the coatings prepared at pH = 6 is due to lower average surface roughness and more dense structure of HAp. It is known that the rougher surfaces are generally less resistant to corrosion, especially when it means that large reflective areas exposed to corrosive media and the valleys retain the corrosive solution which acts as micro-reaction sites and traps corrosion products, promoting the continual growth of pits^56^. The coating prepared at pH = 6 has lower porosity of the coating, which is another reason of its good corrosion resistance. The HAp coatings prepared in solution with pH = 6 proved to be more densely packed with the features having stronger crystallographic orientation (see XRD results). It was reported that the porosity of a coating decreases when it has a more dense grain structure^63,64^. Moreover, compared to the uncoated substrate and regardless of the SBF exposure period, both coatings presented an enhanced *in vitro* ability to form apatite on the surface, indicating the efficiency of the coatings obtained by EDS. The coatings prepared in electrolyte with pH = 6 exhibited some higher biomineralization ability compared with those obtained in electrolyte with pH = 5; after 21 days of SBF exposure have registered an additional apatite mass approximately three times higher than that at 14 days (almost 7 mg in total). *In vivo* subcutaneous implantation of the coated and uncoated samples into the white rats for up to 21 days with following histological studies showed no serious inflammatory process.

Our results demonstrate that the pH of electrolyte greatly influences the *in vitro* and *in vivo* properties of the HAp coatings. Based on the obtained results, it can be concluded that the electrodeposition of HAp in electrolyte with pH = 6 can become a cost effective method for additively manufactured Ti64 implant surface bioactivation.

## Methods

### Metallic sample manufacturing

Titanium Ti64 parts produced by the method of electron beam melting (EBM) were used. The samples were produced by the ARCAM A2 EBM machine (Arcam AB, Mölndal, Sweden) using a powdered alloy (Ti6Al4V ELI) certified for biomedical applications^65^. Powder was purchased from ARCAM AB and has a grain size distribution from 50 to 150 µm. Samples were disc-shaped with the diameter of 20 mm and thickness of 1 mm. All used samples were manufactured in the same batch with the build direction along the flat surface. Manufacturing layer thickness was 70 µm, and standard ARCAM parameter settings for solid Ti64 components were used. All samples were carefully blasted in the ARCAM powder recovery system using a precursor powder. Corresponding design files were prepared using a software package Solid Works (by Dassault Systems) and prepared for printing using an ARCAM Build Assembler. More detailed descriptions of the corresponding procedures can be found in the literature^66,67^.

Electron beam melting process happens in a working chamber under high vacuum (approximately 10^−6^ Bar). This is so-called powder bed process, in which the deposited thin layer of the powder is first semi-sintered by a de-focused beam. After that corresponding sections of the layer are melted by sharply focused high power beam. Than a next layer of the working powder is deposited and sintering-melting process for the next layer is carried on. After completing all layers of the build the process is stopped, and the component surrounded by semi-sintered powder is cooled down. After cooling remaining unused powder is removed in a special blaster chamber (powder recovery system) using precursor powder to avoid component surface contamination by other material^68^. One of the characteristic properties of the EBM processing is the ability to “stack” multiple components allowing their successive manufacturing in the same continuous process. Used samples are disc shaped with a diameter of 10 mm and thickness of 1.5 mm.

### Coating process

The coating of the Ti64 surfaces was performed by the electrochemical deposition using the following protocol:

- before the electrochemical deposition all samples were ultrasonically cleaned in acetone in order to remove small residuals, grease and air bubbles trapped in the metallic scaffold;
- electrochemical deposition of HAp was conducted in a typical three electrode electrochemical cell: the working electrode (WE) was the Ti64 disk; the reference electrode (RE) was a saturated calomel electrode (SCE); the counter electrode (CE) was a platinum foil with 1 cm^2^ surface area;
- electrodeposition was controlled using a Potentiostat/galvanostat (Parstat 4000, Princeton Applied Research, USA), operating in potentiodynamic mode, with an applied potential to WE from −1.4 to 0 V referenced to the SCE, changing at constant rate of 0.2 mV/s.

The electrolyte has been prepared by subsequently dissolving calcium nitrate (Ca(NO_3_)_2_) and ammonium dihydrogen phosphate (NH_4_H_2_PO_4_), both from Sigma-Aldrich (Germany), in ultrapure water. Electrolyte resistivity was 18 MΩ·cm at 25 °C. Two types of electrolyte have been used and prepared in this study as follows: the first electrolyte solution was used as prepared and had a pH = 5; the second one was prepared from the first one by adjusting its acidity to pH = 6 by adding 1 M NaOH. The pH value around 5 was selected because it is known that in the proximal area of the surgical incision of the implant, the pH can drop to low values (~4), mainly due to biomaterial associated infection or can be caused just by the surgical insertion itself, this being a very traumatic experience for the human body^69^. The sample code names and chemical composition of the electrolytes used for electro depositing of the coatings are presented in Table 4. The deposition was performed at 75 °C (±0.5 °C) under continuous electrolyte stirring for approximately 2 h. After the electrodeposition, the samples were rinsed in distilled water and dried at room temperature in a desiccator for 24 h in order to remove the excessive water.

**Table 4:** Sample codification and chemical composition of the electrolyte.

Sample code	Electrolyte – chemical composition		pH
	Ca(NO_3_)_2_·4H_2_O (mM)	NH_4_H_2_PO_4_ (mM)	
HAp/Ti64/pH5	0.61	0.36	5 ± 0.2
HAp/Ti64/pH6	0.61	0.36	6 ± 0.2 (adjusted with 1 M NaOH)

### Physical, chemical and morphological characterization

The morphology and elemental composition of the coated samples have been investigated by a scanning electron microscope (SEM, TableTop 3030PLUS, Hitachi, Japan) equipped with energy dispersive spectrometer (EDS, Quantax70, Bruker, USA). The chemical bonding of the coatings was analysed using the Fourier transform infrared spectrophotometer (FT-IR- 6300, Jasco, Japan) at a resolution of 4 cm^−1^, over the wavelength range 500–4000 cm^−1^ using an universal ATR sampling accessory. The phase composition of the coatings was examined by an X-ray diffractometer (SmartLab, Rigaku, Japan) with CuK_α_ radiation (λ = 1.5405 Å). A detailed scan was done over the 2θ range from 10 to 80° with a scan rate of 0.2°/min. X-ray photoelectron spectroscopy (XPS) was carried out in the near surface region of uncoated Ti64 substrate by an Escalab Xi^+^ spectrometer using AlK_α_ radiation. A 2 keV Ar^+^ ion beam was used for sputter etching of the sample (10 min). The surface morphology was also evaluated by an Atomic Force Microscope (AFM, INNOVA, Veeco, USA), operating in the tapping mode using 10 × 10 μm^2^ area scans. Elemental composition of the SBF solution and corresponding ion concentrations are presented in Table 5 in comparison with the parameters of actual human blood plasma.

**Table 5:** Reagents for SBF preparation and the nominal ion concentration of used SBF (prepared according to^74^) in comparison with human blood plasma (according to^75^).

No.	Simulated Body Fluid (SBF)		Ion	Ion concentration (mM)	
	Reagents	Mass		SBF	Blood plasma
1	NaCl	8.035 gL^−1^	Na^+^	142.0	142.0
			Cl^−^	147.8	103.0
2	NaHCO_3_	0.335 gL^−1^	HCO_3_^−^	4.2	27.0
3	KCl	0.225 gL^−1^	K^+^	5.0	5.0
4	K_2_HPO_4_·3H_2_O	0.231 gL^−1^	HPO_4_^2−^	1.0	1.0
5	1 M HCl	40 cm^3^	—	—	—
6	MgCl_2_·6H_2_O	0.311 gL^−1^	Mg^2+^	1.5	1.5
7	CaCl_2_	0.292 gL^−1^	Ca^2+^	2.5	2.5
8	Na_2_SO_4_	0.072 gL^−1^	SO_4_^2−^	0.5	0.5
9	(HOCH_2_)_3_CNH_2_	6.228 gL^−1^	—	—	—
pH				**7.4**	**7.35 ÷ 7.45**

### Electrochemical tests

The electrochemical behaviour was studied in SBF solution with pH = 7.40, at 37 ± 0.5 °C using a Potentiostat/galvanostat (Parstat 4000, Princeton Applied Research, USA). An area of 1 cm^2^ was exposed to the SBF by placing the samples in a Teflon sample holder (working electrode). All tests were performed on the disc-shaped samples under the same conditions using a typical three-electrode cell with the following set-up: sample as working electrode (WE), a platinum foil used as a counter electrode (CE) and saturated calomel electrode (SCE) as reference electrode (RE). The measurements were repeated at least two times. The open circuit potential (OCP) was monitored during 1 h. The potentiodynamic curves were recorded from −1 Veto +1 V on WE Vs SCE. Corrosion potential (E_corr_) and current density (i_corr_) parameters were determined from the Tafel extrapolations^58^. Polarization resistance (R_p_) was also determined from the Tafel plots at i = 0 as a slope of potential dependence versus current density:

10 $${R}_{p}=\frac{1}{2.3}\cdot \frac{{b}_{a}|{b}_{c|}}{{b}_{a}+|{b}_{c}|}\cdot \frac{1}{{i}_{corr}}$$

The coatings protective efficiency (P_e,_) was calculated from the corrosion parameters determined from the Tafel extrapolation as follows:

11 $${P}_{e}=(1-\frac{{i}_{corr,coating}}{{i}_{corr,substrate}})\cdot 100$$

where i_corr,coatings_ and i_corr,substrate_ are the corrosion current densities of the coating and the substrate, respectively.

The porosity (*P*) was calculated based on Elsener’s empirical equation (Eq. 12)^70^:

12 $$P=(\frac{{R}_{{\rm{p}},\mathrm{substrate}}}{{R}_{{\rm{p}},\mathrm{coating}}})\cdot 10\frac{-|{\rm{\Delta }}{E}_{{\rm{corr}}}|}{\beta {\rm{a}}}$$

where: *R*_p,substrate_ and *R*_p,coating_ are polarization resistance of the substrate and the obtained coatings, respectively; Δ*E*_corr_ is difference between the corrosion potentials of the coatings and the uncoated substrate.

### *In vitro* biomineralization ability tests

Biomineralization ability of the coatings was investigated using *in-vitro* bioactivity assay. With this test, actual biomineralization ability can be predicted not only qualitatively but also quantitatively. This test can be used for a preliminary assessment of the biological interlocking of the coated implants with the surrounding tissue during their biointegration. It is important to mention that this particular test is fast and cost-effective, and could be considered as a good prerequisite before performing cell experiments and *in vivo* (laboratory animals) biocompatibility tests, which are rather expensive and can need lengthy approval procedures.

The coated and uncoated samples of Ti64 were immersed in SBF for 1, 3, 7, 14 and 21 days at human body temperature (37 ± 0.5 °C) in a Memmert IF 55 incubator (Memmert GmbH, Germany). The testing SBF media was renewed daily in order to prevent chemical exhaustion and to eliminate the possibility of bacteria/microorganisms growth. The mass of apatite formed on the exposed surface was monitored using an analytical balance having an accuracy of 0.01 mg. The weight variation of the formed apatite on the surface was determined using the following equation:

13 $${\rm{\Delta }}m={{\rm{m}}}_{{\rm{f}}}-{{\rm{m}}}_{{\rm{i}}}\quad ({\rm{mg}})$$

where: Δm – is the apatite mass formed on the surface, m_i_ and m_f_ are the sample weight before and after exposure to SBF, correspondingly.

After removing the samples from SBF media and before weighting, all samples have been rinsed in distilled water and dried in the desiccator for 24 h in order to remove the water from their structure. The *in vitro* bioactivity results for the coated and uncoated samples are presented in terms of evolution of apatite mass formed on the material surface.

### Animal experiments

Biocompatibility assessment *in vivo* was carried out on 20 white nonlinear male rats weighing 200–260 g. Experimental animals were divided into two groups with 10 animals each. Group 1 (control group) consisted of animals that were subjected to subcutaneous implantation of uncoated Ti64 samples in the interscapular region. Group 2 (experimental group) consisted of animals that were subjected to subcutaneous implantation of coated samples in the interscapular region.

Implantation tests were performed in compliance with the principles of bioethics, rules of Good Laboratory Practice (GLP), and conventions for the protection of animals used in experiments and for other scientific purposes (adopted by the Council of Europe in 1986) in accordance with the order of the Ministry of Health of the Russian Federation no. 267 from June 19, 2003 “Approval of the Rules of Good Laboratory Practice”. This study is compliant with the ethical standards set out in the international guidelines for the care and use of laboratory animals^71–73^. The protocol of implantation was approved by the ethic committee of Saratov State Medical University named after V. I. Razumovsky 19 December, 2016 (Protocol no. 4).

A combination of Zoletil (VirbacSanteAnimale, France) at a dose of 0.1 ml/kg and Xylazine (Interchemie, Netherlands) at a dose of 1 mg/kg was administered intramuscularly to all animals in order to achieve anaesthesia 5 min prior to surgical manipulations. After rat anaesthetization, the dorsal skin in the interscapular area was carefully shaved without any detectable or visible damage. Linear skin incision of 1.5 cm was made after the surgical field had been treated with 70% ethanol. Subcutaneous pocket with the size of approximately 1.5 × 1.5 cm^2^ was formed by blunt dissection with the help of forceps jaws. A sample of porous Ti64 alloy was placed in subcutaneous pocket and a wound was sutured by non-absorbable monofilament “Rezopren” M2 (3/10) with the stitching needle of 27 mm (RESORBA Medical GmbH, Germany). After suturing, the wound was treated with 70% ethanol.

To perform morphological analysis, the rats of the control and experimental groups were sacrificed on the 21^st^ day by anaesthetic overdose. The whole block of tissue including the sample was dissected from the implantation region. Soft tissues were separated from the sample, fixed in 10% neutral formalin (BioVitrum, Russia), dehydrated in increasing strength ethanol solution series, embedded in paraffin wax, and sectioned at 5–7 µm. Sections were stained with Mayer’s Haematoxylin and Eosin solution (BioVitrum, Russia) and mounted with Bio-Monht mounting medium (BioOptica, Italy). Imaging analysis of preparations was performed using AxioImager Z2 microscope (Carl Zeiss, Germany) and included evaluation of the structure, cellular composition and state of microvasculature in soft tissues surrounding the sample. The cell number of each type (cell population) was counted in five fields of view in hypodermal connective tissue.

### Statistical analysis of the data

All obtained data were indicated as average ± standard deviations (SD) and for each sample the paired Student’s t-test was used. Statistical significant value was determined as p < 0.05.

### Future possibilities

Though present research was not targeting advanced studies on the porous (lattice) Ti64 structures, initial tests were conducted. These tests indicate that electrodeposition is effective with certain geometries of the lattice structures, and can provide adequate HAp coatings with good corrosion resistance and *in vitro* biomineralization. Initial *in vivo* tests of the ED coated porous samples are also quite promising. Thus wider studies of the Ti64 samples containing both porous and solid elements (most indicative for the realistic modern implant constructions) should be carried out.

## Conclusions

As conclusions, the following can be drawn:

- the pH adjustment from 5 to 6 has led to a difference in morphology of the obtained coatings: the coatings prepared at a pH = 5 were uniform and consisted in dense nano-walls and nano-flakes with pore dimensions of ~350 nm and a high surface-to-volume ratio, and the coatings prepared at a pH = 5 predominantly consist of needle like structures with dimensions in the nanometric scale (~30 nm);
- the corrosion resistance of coated samples in SBF media was significantly better compared to Ti64 ones, samples prepared at a pH = 6 showing smallest corrosion current density and the higher polarization resistance, which indicates a good corrosion resistance;
- both coated samples have shown high protection efficiency to the corrosion attack, however the ones electrodeposited at pH = 6 (92.9%) were performing better as compared to the ones obtained at pH = 5 (88.9%);
- during the *in-vitro* bioactivity tests in the simulated body fluid both HAp/Ti64/pH6 and HAp/Ti64/pH5 coated samples have shown good mineralization ability, with surfaces entirely covered with newly formed apatite layers;
- corresponding mass gain per surface unit of the HAp/Ti64/pH6 and HAp/Ti64/pH5 samples was 5.5 and 4.71 mg/cm^2^ after 21 days of exposure to SBF, indicating a good biomineralization ability for the coating prepared at pH = 6;
- *in vivo* tests were performed on the coated but not additionally *in-vitro* biomineralized HAp/Ti64/pH6 and Ti64 samples in white rats; the results of histological studies indicated the absence of local inflammatory process on the 21^st^ day after subcutaneous implantation of both types of experimental samples proving biocompatibility of the samples and showing no negative immune response *in vivo*.

According to the conducted research, both HAp ED coatings showed higher potential for the biointegration as compared to the as-manufactured Ti64 ones. Though both ED coated samples have similar properties with *in vitro* biomineralization and *in vivo* tests, the ones prepared in the electrolyte with pH = 6 revealed a superior effect. Thus, electrodeposition of HAp can become a cost effective method to fully cover implantable medical devices with complex geometry, rough and/or porous surface prepared by the additively manufactured Ti64 implant surface bioactivation.
